# Loss of the p53 transactivation domain results in high amyloid aggregation of the Δ40p53 isoform in endometrial carcinoma cells

**DOI:** 10.1074/jbc.RA119.007566

**Published:** 2019-04-26

**Authors:** Nataly Melo dos Santos, Guilherme A. P. de Oliveira, Murilo Ramos Rocha, Murilo M. Pedrote, Giulia Diniz da Silva Ferretti, Luciana Pereira Rangel, José A. Morgado-Diaz, Jerson L. Silva, Etel Rodrigues Pereira Gimba

**Affiliations:** From the ‡ Instituto Nacional de Câncer, Coordenação de Pesquisa, Programa de Oncobiologia Celular e Molecular, Rio de Janeiro, Brazil,; the § Instituto de Bioquímica Médica Leopoldo de Meis, Instituto Nacional de Ciência e Tecnologia de Biologia Estrutural e Bioimagem, Centro Nacional de Biologia Estrutural e Bioimagem, Universidade Federal do Rio de Janeiro, Rio de Janeiro 21941902, Brazil,; the ¶ Department of Biochemistry and Molecular Genetics, University of Virginia, Charlottesville, Virginia 22908,; the ‖ Faculdade de Farmácia, Universidade Federal do Rio de Janeiro, Rio de Janeiro 21941-170, Brazil,; the ** Universidade Federal Fluminense, Instituto de Humanidades e Saúde, Departamento de Ciências da Natureza, Rio de Janeiro 28895-532, Brazil, and; the ‡‡ Universidade Federal Fluminense, Programa de Pós Graduação em Ciências Biomédicas-Fisiologia e Farmacologia, Niterói 24210-130, Brazil

**Keywords:** protein aggregation, p53, cancer biology, amyloid, protein misfolding, endometrium carcinoma, p53 isoforms, p53 transactivation domain (TAD)

## Abstract

Dysfunctional p53 formation and activity can result from aberrant expression and subcellular localization of distinct p53 isoforms or aggregates. Endometrial carcinoma (EC) is a cancer type in which p53 status is correlated with prognosis, and *TP53* mutations are a frequent genetic modification. Here we aimed to evaluate the expression patterns of different p53 isoforms and their contributions to the formation and subcellular localization of p53 amyloid aggregates in both EC and endometrial nontumor cell lines. We found that full-length (fl) p53 and a truncated p53 isoform, Δ40p53, resulting from alternative splicing of exon 2 or alternative initiation of translation at ATG-40, are the predominantly expressed p53 variants in EC cells. However, Δ40p53 was the major p53 isoform in endometrial nontumor cells. Immunofluorescence assays revealed that Δ40p53 is mainly localized to cytoplasmic punctate structures of EC cells, resembling solid-phase structures similar to those found in neurodegenerative pathologies. Using light-scattering kinetics, CD, and transmission EM, we noted that the p53 N-terminal transactivation domain significantly reduces aggregation of the WT p53 DNA-binding domain, confirming the higher aggregation tendency of Δ40p53, which lacks this domain. This is the first report of cytoplasmic Δ40p53 in EC cells being a major component of amyloid aggregates. The differential aggregation properties of p53 isoforms in EC cells may open up new avenues in the development of therapeutic strategies that preferentially target specific p53 isoforms to prevent p53 amyloid aggregate formation.

## Introduction

Endometrial carcinoma (EC)^3^ is the most common gynecological malignancy in the Western world. It is traditionally classified as type I and type II tumors (1), which is based on clinical, endocrine, histopathological, genetic, as well as molecular features.

More recently, the Cancer Genome Atlas Research network classified them into four molecular subgroups (2). Mutations at *TP53* are present in 5–12% of all type I EC cases, as opposed to type II EC, in which *TP53* mutations are the most common genetic modification (3). It has also been described that p53 overexpression in EC tumors is strongly predictive of recurrent EC and mostly not correlated with TP53 mutations (4). Moreover, it has been revealed that EC tumors presenting extensive copy number alterations and TP53 mutations are highly aggressive (2). Thus, TP53 genetic modifications are key features in EC biology.

The human *TP53* gene encodes a nuclear protein that generally behaves as a tumor suppressor and responds to stress conditions to induce cell cycle arrest, senescence, or programmed cell death (5). The roles of p53 are controlled by transcriptional and translational mechanisms, protein stability, and subcellular localization. In particular, the regulation of p53 subcellular localization depends on factors that influence its nuclear import and export, subnuclear localization, and cytoplasmic tethering and sequestration (6). Notably, cytoplasmic expression provides additional roles to p53, such as modulating apoptosis via a transcription-independent action (7), autophagy, metabolism, oxidative stress, and drug response (8). Cytoplasmic inclusions of p53 have also been correlated with sequestration of p53 as large protein amyloid aggregates (9).

Somatic *TP53* mutations are the most frequent in most human cancers and eliminate its tumor suppressor functions and promote oncogenic properties (5). It is often stated that 50% of cancers have mutated or inactivated p53. However, the real number is probably much higher when the involvement of the entire p53 pathway in tumorigenesis is considered. In tumors in which *TP53* is not mutated, p53 itself or its signaling can be inactivated by posttranscriptional and posttranslational modifications, subcellular localization, and interaction with other proteins (10). In tumor cells, a dysfunctional p53 protein often presents an aberrant misfolded and inactive conformation, which accumulates and aggregates to form amyloid-like oligomers and fibrils, related to impairment of p53 roles (11–13). Accordingly, p53 aggregation may be a crucial step in tumor progression. Our group and others have suggested that the formation of mutant p53 aggregates is associated with loss-of-function, dominant-negative, and gain-of-function effects and that these features seem to be correlated with its prion-like behavior (11–15). Intriguingly, WT p53 aggregation has also been reported in high-grade serous ovarian carcinoma cancer cells exhibiting cancer stem cell properties, which is associated with p53 loss of function and platinum resistance (16). Thus, it seems that not only mutant but also WT p53 can undergo aggregation and dysfunction.

**Figure 1.**
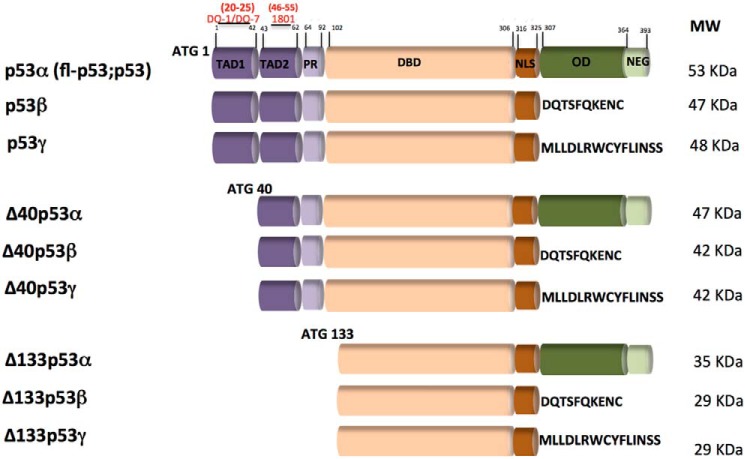
**Schematics of the p53 isoform domains.** The canonical p53 (p53α) or full-length (fl-p53 or p53) isoform has two transactivation domains (TAD1, aa 1–42, and TAD2, aa 43–63), a proline-rich domain (*PR*, aa 64–92), a DBD (aa 102–306), a nuclear localization domain (*NLS*, aa 316–325), an OD (aa 307–355), and a negative regulation domain (*NEG*, aa 364–393). The predicted molecular mass in kilodaltons of each isoform is shown on the right. Above the fl-p53 structure are indicated, in *red*, the domain regions to which the DO-1, DO-7, and 1801 anti-p53 antibodies bind. *ATG*, translation initiation codon used to initiate each N-truncated p53 protein isoform. Adapted from Ref. 17.

The human *TP53* gene expresses at least 12 p53 isoforms as a result of distinct molecular mechanisms such as alternative splicing, alternative promoter, or alternative initiation codon use (17) (Fig. 1). Each p53 variant can be combined into three distinct C-terminal forms (α, β, and γ). In the C terminus domain, the α isoforms contain an oligomerization domain (OD), whereas the β and γ isoforms include novel amino acid residues instead of an OD. The fl-p53, p53β, and p53γ proteins contain the conserved N-terminal transactivation domain (TAD). Δ40p53 isoforms are truncated proteins resulting from alternative splicing of exon 2 and/or alternative initiation of translation at ATG-40. The Δ40p53 protein lacks the conserved N-terminal TAD1 but still contains TAD2. Δ133p53 isoforms are truncated p53 proteins with the entire TAD and part of the DBD deleted. Translation is initiated at ATG-133. These isoforms can modulate p53 transcriptional activity in the absence of genomic alterations and are capable of altering p53 function besides being differentially expressed in tumor and nontumor cells (18). Most of these p53 variants share a common DNA-binding domain (DBD) with three different N-terminal domains. By convention, p53α corresponds to the canonical or full-length (fl) p53. In addition to these isoforms, Δp53 variants lack N-terminal domains (18). Although these p53 isoforms have been hypothesized to aggregate differently in cancer cells (19), experimental data showing the contribution of p53 isoforms to protein oligomer aggregation are still lacking. The N-terminal TAD is intrinsically disordered and has been shown recently to interact with the DNA-binding surface of the DBD through primarily electrostatic interactions, resulting in inhibition of binding of nonspecific DNA (20).

Given the key contributions of TP53 genetic modifications in EC etiopathogenesis, the p53 aggregation potential, and related functional roles of p53 isoforms, this work aimed to evaluate the expression profiles of some p53 isoforms in EC tumor and endometrial nontumor cell lines. We then investigated the aggregation potential and subcellular localization of differentially expressed p53 isoforms, mainly focusing on fl-p53 and Δ40p53 variants. Moreover, we also compared the *in vitro* aggregation properties of recombinant p53 constructs to test whether the intrinsically disordered p53 TAD modulates its DBD aggregation.

## Results

### Transcriptional profile of p53 isoforms in endometrial carcinoma

**Figure 2.**
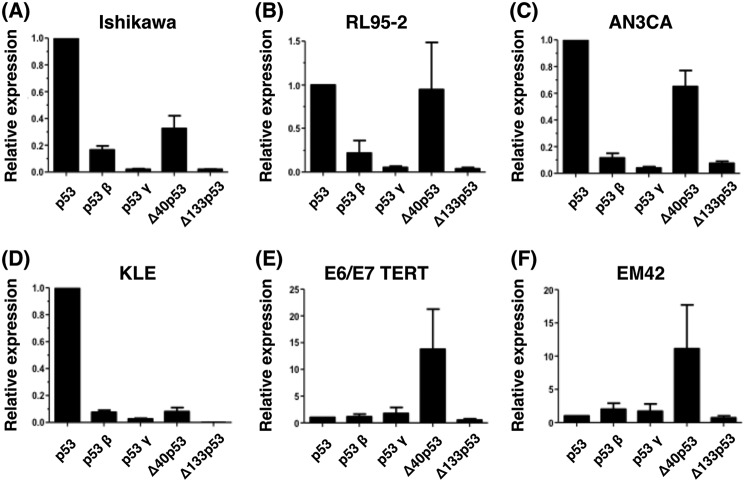
**Transcript levels of p53 isoforms in endometrial tumor and nontumor cell lines.** *A–F*, relative transcript levels of fl-p53 (*p53*), p53β, p53γ, Δ40p53, and Δ133p53 isoforms were determined using quantitative real-time PCR and isoform-specific oligonucleotide pairs (shown in Table 1). The β-actin gene was used as a normalization control. Data are representative of three independent experiments, using duplicates in each assay. We tested four tumor endometrial cell lines (Ishikawa (*A*), RL95-2 (*B*), AN3CA (*C*), and KLE (*D*)) and two endometrial nontumor cell lines (E6/E7/TERT (*E*) and EM42 (*F*)). The expression levels of fl-p53 (*p53*) were used as a reference to calculate the relative expression level of each p53 isoform.

We investigated the expression of five p53 isoforms: fl-p53 (p53), p53β, p53γ, Δ40p53, and Δ133p53 (Fig. 2 and Fig. S1) in EC tumor (Ishikawa, RL95–2, AN3CA, and KLE) and nontumor cell lines (E6/E7/TERT and EM42), and determined their putative subcellular localization and presumed aggregation potential.

We found that transcripts coding for the tested p53 isoforms are differentially expressed between EC tumor and nontumor cell lines (Fig. 2 and Fig. S1). Markedly, fl-p53 followed by Δ40p53 are the major p53 isoforms expressed in EC cells (Fig. 2, *A–D*). Especially in cell lines representative of type I EC tumors (Ishikawa, RL95–2, and AN3CA), Δ40p53 was expressed at higher levels than the remaining shorter p53 variants (Fig. 2). Δ40p53 variant was also the major isoform expressed in EC nontumor cells (Fig. 2, *E* and *F*).

### Protein expression of p53 isoforms in endometrial carcinoma

We also characterized the protein expression levels of the fl-p53 and Δ40p53 isoforms using immunoblotting of these same cell lines (Fig. S2). Because specific antibodies against Δ40p53 cannot be produced, its protein levels were determined only indirectly with a combination of anti-p53 antibodies. Δ40p53 expression was evaluated using the anti-p53 monoclonal antibodies DO-1, DO-7, and 1801, as described before (17). These preliminary findings showed that, when fl-p53 expression was analyzed using the DO-1 or DO-7 antibodies, which only detect the fl-p53 variant (with a molecular mass of around 53 kDa), higher levels of fl-p53 were observed compared with those found in the nontumor cell lines E6/E7/TERT and EM42 (Fig. S2, *A* and *B*), as generally observed at the transcriptional level (Fig. S1). Similar data have also been found using the 1801 anti-p53 antibody, which detects both the fl-p53 (53 kDa) and the Δ40p53 (47 kDa) variants (Fig. S2*C*), as described previously (17). Notably, in nontumor cells, lower levels of the Δ40p53 protein were observed compared with EC tumor cells, even though the corresponding gene transcript was the most abundant in nontumor cells (Fig. 2 and Figs. S1 and S2). Therefore, transcriptional and supporting protein analyses of these p53 isoforms provide evidence that mainly fl-p53 and Δ40P53 display differential expression patterns in EC tumor and nontumor cells and that the transcript levels of these isoforms display a tendency to correspond to the respective protein levels.

### Aggregation potential of p53 isoforms in endometrial carcinoma

**Figure 3.**
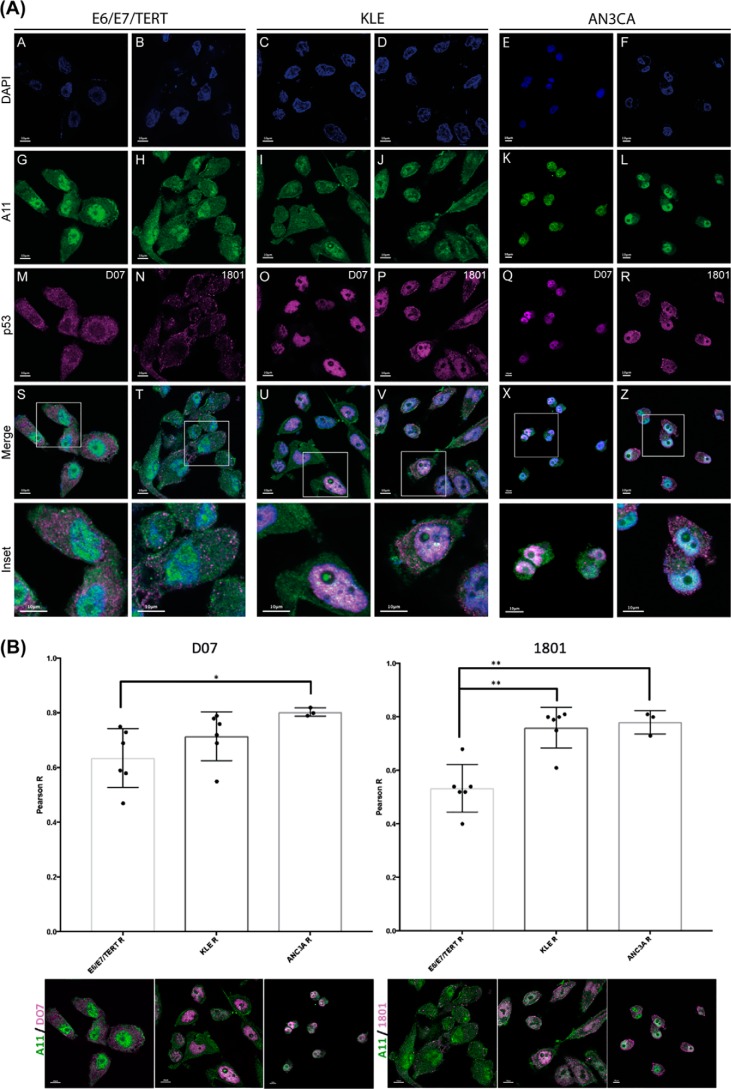
**The Δ40p53 isoform is a major component of cytoplasmic amyloid aggregates in EC cell lines.** *A*, immunofluorescence analysis of E6/E7/TERT (an endometrial nontumor cell line) and KLE and AN3CA (endometrial tumor cell lines) stained with A11, DO-7, and 1801 anti-p53 primary antibodies and secondary goat anti-mouse IgG/Alexa 546 and goat anti-mouse IgG/Alexa 488 antibodies. The A11 anti-amyloid oligomer antibody (*green*) was used to detect amyloid oligomer aggregates, and DO-7 and 1801 anti-p53 antibodies were used to detect, respectively, fl-p53 and Δ40p53 (*magenta*) isoforms. Cells were counterstained with DAPI to identify nuclei (*blue*). *Scale bars* = 10 μm. *B*, quantification of co-localization between A11 and anti-p53 antibodies. Quantification of co-localization between A11 (*green*) and DO-7 (*magenta*) or A11 (*green*) and 1801 (*magenta*) antibodies in the E6/E7/TERT, KLE, and ANC3A cell lines is shown, respectively, on the *left* and *right. Dots* represent one quantified image with at least 5–10 cells (*n* > 15 cells for all studied conditions). The images in *B* are the same images as in *A* but without the nuclei (DAPI staining). The immunofluorescence images from which the quantification was performed are shown below each bar. *Scale bars* = 10 μm. Statistical significance was set as *, *p* < 0.05 and **, *p* < 0.005 using nonparametric Kruskal–Wallis and Dunn tests. Images were visualized using a confocal microscope (FV10i-O, Olympus).

We next considered whether fl-p53 and Δ40p53 contribute differently to the formation of p53 amyloid aggregates and to the isoforms' subcellular localization. We performed immunofluorescence analysis of E6/E7/TERT, KLE, and AN3CA cells using the A11 amyloid oligomer-specific antibody, DO-7, and 1801 anti-p53 antibodies (Fig. 3*A*).

Differential staining patterns between these two antibodies would indicate Δ40p53-specific staining in these cells. A11 antibody staining exhibited a diffuse pattern throughout the cells (Fig. 3, *A* and *G–L*, *green*). As shown by the DO-7 staining pattern, fl-p53 presented a differential staining pattern shifting from predominantly cytoplasmic staining in E6/E7/TERT EC nontumor cells to a cytoplasmic and mainly nuclear staining pattern in KLE and AN3CA EC cells (Fig. 3, *A*, *M*, *O*, and *Q*). Notably, as evidenced by 1801 antibody staining, we found a very particular pattern of p53 punctate cytoplasmic structures in all three cell lines analyzed (mostly in EC tumor cells) in addition to nuclear p53 staining in KLE and AN3CA EC cells (Fig. 3, *A*, *O–R*, and *U–X*). Taking into consideration the different affinities of DO-7 and 1801 antibodies (Fig. S2) for fl-p53 and Δ40p53, respectively, we might infer that their differential staining patterns possibly reflect Δ40p53-specific staining. A concentration of puncta was observed in the regions between cells and also under the plasma membrane (Fig. 3, *A*, *T*, *V*, and *X* and the corresponding *insets*). Through co-localization evaluation and correlation analysis, we further assessed the interaction between p53 isoforms and amyloid aggregates. Although the Δ40p53 staining patterns were similar among the cell lines analyzed, we found a higher number of 1801-stained puncta and a higher degree of co-localization between 1801- and A11-stained amyloid aggregates in EC cells (Fig. 3, *A*, *T*, *V*, and *X* and *insets*; quantification is shown in Fig. 3*B*). However, scattered cytoplasmic and nuclear DO-7–stained patterns were mainly observed in nontumor and EC tumor cell lines, respectively (Fig. 3, *A*, *M*, *O*, *Q*, *S*, *U*, and *W* and *insets*).

### The transactivation domain of p53 prevents its aggregation

**Figure 4.**
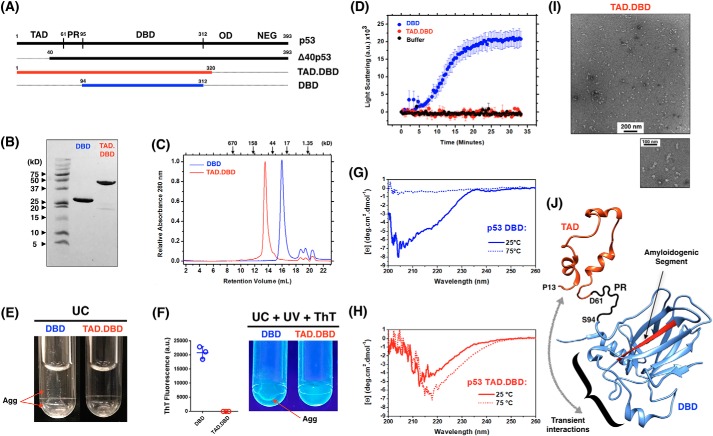
**The transactivation domain is a negative regulator of p53 aggregation.** *A*, schematics of the TAD.DBD (*red*) and DBD (*blue*) constructs recombinantly expressed in this work. *B*, 20% SDS-PAGE stained with Coomassie Brilliant Blue showing purified proteins. *C*, analytic size-exclusion chromatography of both constructs monitored by absorbance at 280 nm. *D*, time course measuring the light scattering of p53 TAD.DBD and DBD at 37 °C. Results are shown as the average of three independent experiments. *E*, UC tubes showing DBD aggregation and a clear solution of the TAD.DBD construct. *F*, UV light illumination of ThT-bound DBD aggregates (*Agg*) after incubation at 37 °C for 40 min and UC. ThT fluorescence was quantified and is expressed as the average ± S.E. (*n* = 3). *G* and *H*, far-UV CD spectra of DBD (*G*) and TAD.DBD (*H*) at room temperature and at 75 °C. *I*, transmission EM of TAD.DBD species formed after heating the sample to 75 °C. *J*, atomic model representation of the TAD (PDB code 2L14, residues 13–61) and the DBD (PDB code 2FEJ, residues 94–297) to illustrate the negative regulation of the TAD segment over p53 DBD aggregation. The amyloidogenic segment of the DBD is colored *red. PR*, proline region; *NEG*, negative regulatory domain. The *curly bracket* shows the p53 DNA-binding motif in which the TAD potentially interacts according to Ref. 11.

**Figure 5.**
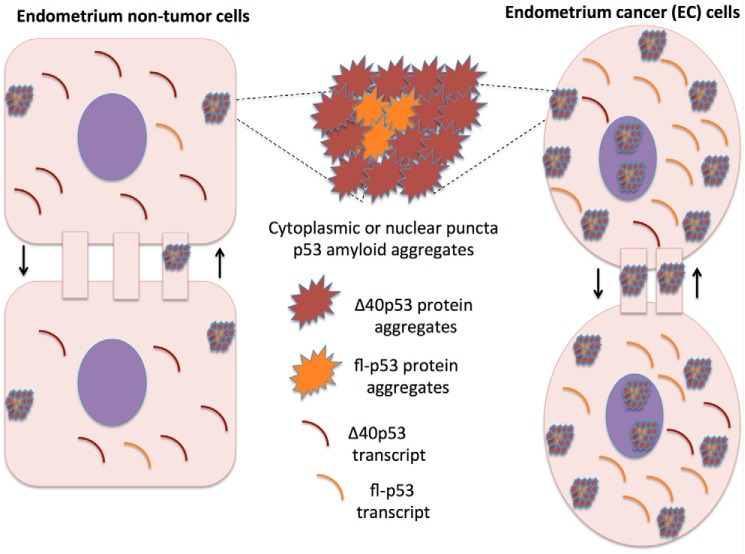
**The Δ40p53 isoform in EC cells.** The schematic shows Δ40p53 as a modulator of p53 tumor suppression and oncogenic activities in EC cells.

As an attempt to explain what triggers the higher amyloid aggregation of the Δ40p53 isoform in EC cells, we hypothesized whether the missing N-terminal residues of this isoform (residues 1–40) from the TAD would favor aggregation. To do so, we recombinantly expressed a construct containing p53 residues 1–320 comprising the p53 TAD, residues 1–61, and the DBD (Fig. 4*A*, *red*). As shown previously, we used the p53 DBD construct (residues 94–312) as a standard for p53 aggregation (11) and (Fig. 4*A*, *blue*). After purification of both constructs (Fig. 4*B*) and analytical size-exclusion chromatography verification (Fig. 4*C*), we measured the time course of both proteins by light scattering at physiological temperature (Fig. 4*D*). Notably, the construct containing the full-length TAD and the DBD region revealed negligible scattering compared with the DBD alone, suggesting that no higher-order aggregates were formed. Ultracentrifugation (UC) after incubating both constructs at physiological temperature for 40 min showed aggregation for the DBD and a clear solution for the DBD construct containing the TAD segment (Fig. 4*E*). Additionally, the aggregation of the DBD construct revealed weak thioflavin T (ThT) binding against UV light (Fig. 4*F*). To further explore the aggregation potential of the TAD within the longer construct, we increased the temperature of both constructs from 25 °C to 75 °C using a constant rate of 1 °C/min. By monitoring the far-UV CD spectra at 25° and 75 °C, the DBD revealed the typical behavior of loss of secondary structure, followed by aggregation and protein precipitation (Fig. 4*G*). Indeed, precipitation was clearly visible at the end of the measurement, similar to what is shown in Fig. 5*E*. Surprisingly, the TAD.DBD maintained a clear solution even after achieving 75 °C, and the CD spectrum was consistent with increased content of β-strands (Fig. 4*H*). These observations support the formation of soluble oligomers, later confirmed by negative staining transmission EM (Fig. 4*I*). Altogether, the data show how the TAD negatively affects the aggregation behavior of p53. It slows down the kinetics of p53 aggregation in such a way that it allows structural reorganization to form ordered oligomeric species.

## Discussion

This work aimed to evaluate the expression patterns and aggregation properties of p53 isoforms in EC and endometrium nontumor cells and the correlation of these patterns with p53 aggregation potential in these cells. We found that although the fl-p53 transcript is the predominantly expressed p53 transcript in EC cells, followed by Δ40p53, the latter is the major component of p53 amyloid aggregates in EC cells. Once Δ40p53 was the major component of p53 cytoplasmic aggregates in EC cells, we propose here that Δ40p53 is a key modulator of p53 tumor suppression and oncogenic activities in EC cells (Fig. 5), as shown in hepatocellular carcinoma, melanoma, and breast tumors (21), in which this isoform presents a dominant-negative role over fl-p53. Our results also suggested that Δ40p53 may be mainly sequestered in cytoplasmic amyloid aggregates, similar to those reported previously to either modulate p53-mediated tumor suppressor roles or its oncogenic activities (22). The presence of these structures in cellular projections between adjacent cells suggests that they can be transported from cell to cell, as reported elsewhere (11, 12, 15). Δ40p53 may modulate p53 aggregation properties and EC progression, in which p53 genetic alterations are related to a poor prognosis (2).

Few studies have described the expression patterns of N-terminally truncated p53 isoforms, including the Δ40p53 variant, in human carcinomas and corresponding nontumor samples. The Δ40p53 isoform lacks the first 40 amino acids encoding the first TAD of p53, the activating phosphorylation sites, and the Mdm2-binding site, the primary regulator of p53 degradation (23). This isoform can result from alternative translation initiation in exon 4 or from alternative splicing of intron 2 and is typically expressed during early embryogenesis, associated with less differentiated and proliferative cells, and generally not expressed in the corresponding adult tissues (21).

The ability of the p53 TAD to inhibit aggregation of the DBD (Fig. 4) could be an explanation for the high Δ40p53 propensity to form amyloid aggregates in the cytoplasm of EC cells. As shown recently by Krois et al. (20), the p53 TAD would directly interact with segments of the p53 DNA-binding site through electrostatic interactions. These authors proposed that these interactions are dynamically transient and would modulate the binding of p53 to DNA (20). It is noteworthy that the p53 DNA-binding motif is not in the vicinity of the previously reported aggregation-prone p53 segment (14, 15) (Fig. 4*J*). However, we showed, by molecular dynamics simulations, that the p53 DBD presents labile segments with an enhanced tendency of exposed backbone hydrogen bonds, a condition that may explain its structural instability at near-physiological temperature and the average-weighted population of molten globule-like species prone to aggregation (24, 25). With that, we tempted to speculate that transient interactions of the TAD within the DNA-binding motif may help to provide overall p53 structural integrity, narrowing the distribution of molten globule-like species, the ones shown previously to be pre-amyloidogenic. Our findings supported the formation of soluble oligomers by TAD.DBD, later confirmed by negative staining transmission EM (Fig. 4*I*), implying that TAD negatively affects the aggregation behavior of p53.

In the context of cancer, our group recently overviewed the pathological aggregation of misfolded p53 in malignant tumors. It has been found that p53 mutants exert a dominant-negative regulatory effect on WT p53 by converting WT p53 into aggregated species, acquiring a gain-of-function (GoF) phenotype and the loss of its tumor suppressor roles. This prion-like behavior of oncogenic p53, as observed for neurogenerative diseases, provides an explanation for its dominant-negative and GoF properties, including the high metastatic potential of cancer cells carrying p53 mutations (11–13). In the context of our current data, isoforms that lack the TAD domain could then modify p53 aggregation properties, similarly to p53 oncogenic mutants, possibly presenting dominant-negative and GoF properties.

We recently found that RNA can modulate the aggregation of p53 DBD and fl-p53 (26). Low RNA:protein ratios resulted in more aggregation than high RNA:protein ratios. This could further explain the higher tendency of Δ40p53 to aggregate in the cytoplasm, where there is lower concentration of RNA. It has been proposed recently that prion-like RNA binding proteins, such as TDP-43 or fused in sarcoma, form solid pathological aggregates in the cytoplasm because low RNA:protein ratios promote phase separation into liquid droplets (27). This could be the case with Δ40p53, which lacks part of the TAD, preventing interaction with nonspecific nucleic acids (20). Recent molecular dynamics data also corroborate our findings, in which it has been reported that the Δ133p53β and Δ160p53β isoforms are much less stable than WT p53β. Moreover, these authors demonstrated that the Δ133p53β dimer could form a relatively stable complex with p53-specific DNA (28).

Several lines of evidence have shown the dominant-negative behavior of N-terminally truncated p53 and p73 isoforms against its full-length counterparts (23, 29, 30). Dominant-negative events are frequently explained when mutated or truncated proteins somehow interfere with the activities of the full-length protein. To explain that, proposed hypotheses include promoter competition and heterocomplex formation (31). It has been shown previously that Δ40p53 is able to form heterotetramers with WT p53 and suppress MDM2-mediated p53 degradation (32). Further, Δ40p53 has been shown to activate gene expression through the second TAD domain (residues 43–63) and has shown a dominant-negative effect toward WT p53, inhibiting p53 transcriptional activity and p53-mediated apoptosis (23, 32). It is worth mentioning that the p53 TAD domain is an essential element for p53 tumor suppressor activities and regulation by posttranslational modifications. Upon DNA damage, a cascade of phosphorylation occurs within the TAD, including Ser-15, Thr-18, and Ser-20, a situation that facilitates p53-MDM2 dissociation and p300/CTB binding for transcriptional activity (33, 34). Indeed, because the first TAD is lacking in Δ40p53, a profound deregulation effect is expected in these cells, especially concerning changes in transcriptional activity and the potential dominant-negative activity toward the full-length protein. Based on the p53 and Δ40p53 transcriptional levels observed in our study, we were tempted to speculate that these are possible mechanisms occurring in EC cells. More interesting, the presence of Δ40p53 aggregates in these cells raises the relevant question of whether gain-of-function effects would also participate in this kind of tumor. These are important aspects awaiting further investigation.

Our immunofluorescence data indicate that mainly cytoplasmic p53 amyloid oligomers may be prevalent in EC cell lines and that these aggregates mostly contain the Δ40p53 isoform. As stated before, fl-p53 and Δ40p53 form heterocomplexes. Because Δ40p53 lacks an Mdm2-binding site, these heterocomplexes escape Mdm2-mediated degradation and therefore can accumulate (35). These characteristics are also consistent with our Δ40p53 results, which is the second major p53 variant in EC cells and the dominant variant in endometrial nontumor cells, being the main component of p53 amyloid aggregates in these cells. Moreover, it has been described that Δ40p53 possesses a p53 conformation that is associated with a more active state and that Δ40p53 can also alter the posttranslational modification profile of fl-p53 (36). Posttranslational modifications at the N terminus of fl-p53, for instance, might increase the recruitment of transcriptional co-activators such as p300 and PCAF and thus be responsible for increased promoter-binding capacity of the heterocomplexes. Thus, we suggest that mainly cytoplasmic and also nuclear amyloid aggregates containing mostly the Δ40p53 variant could be a strategy to efficiently modulate p53 tumor suppressor and oncogenic activities in EC cells. Cytoplasmic structures mainly containing Δ40p53 amyloid aggregates could be related to dysfunctional p53 related to EC progression, a hypothesis that should be validated by further studies. In addition, physical interaction between the Δ40p53 and amyloid aggregates and differential binding relative to fl-p53 should be further validated by other approaches, such as immunoprecipitation.

We provide the first experimental evidence that distinct p53 isoforms can differently contribute to misfolded p53 aggregate states in tumor cells (Fig. 5), especially in EC cells, and possibly in other tumors in which p53 is functionally deregulated. Our *in vitro* work (Fig. 4) clarifies the high propensity of Δ40p53 aggregation in EC cells (Fig. 4*J*). Targeting mutant p53 aggregation has been proposed as a novel strategy against cancer, especially to nullify the devastating gain-of-function effects. Our data support the notion that the Δ40p53 variant can be specifically targeted as a strategy to prevent formation of p53 amyloid aggregates in which this isoform is the major component.

## Experimental procedures

### Cell lines and culture conditions

The endometrium tumor (Ishikawa, RL-95–2, AN3CA, and KLE) and nontumor (E6/E7/TERT and EM42) cell lines were kindly provided by Dr. Tim Hui-Ming Huang and Dr. Ya-Ting Hsu (University of Texas, San Antonio, TX). Cell lines were cultured in DMEM or DMEM/F12 1:1 (Nutrient F-12 Ham, Thermo Fisher Scientific, Waltham, MA) containing 10% FBS and 1% penicillin and streptomycin.

### Quantitative real-time PCR and oligonucleotide sequences

The transcript levels of each p53 isoform was determined using quantitative real-time PCR. Total RNA was extracted using the RNeasy Mini Kit (Qiagen, Hilden, Germany), and complementary DNA synthesis was performed using 1 μg of total RNA. The oligonucleotides primers used for these assays and amplification conditions are shown in Table 1.

**Table 1:** **Oligonucleotide sequence for p53 isoforms** Relative expression levels were calculated using the ΔΔCT method. Cell cycling conditions were as follows: initial incubation at 50 °C for 2 min; followed by 94 °C for 3 min and 34 cycles of 94 °C for 1 min, 58 °C for 1 min, and 72 °C for 1 min; followed by a final incubation at 72 °C for 10 min. Melting curve analysis was performed at 95 °C for 5 s, 50 °C for 5 s, and 95 °C for 5 s.

P53 isoform	Forward primer	Reverse primer
p53α/(fl-p53)	5′-ATG GAG CAG CCG CAG TCA GAT-3′	5′-AAT GTC AGT CAG GCC CTT CTG TC-3′
p53β	5′-GCG AGC ACT GCC CAA CA-3′	5′-GAA AGC TGG TCT GGT CCT GAA-3′
p53γ	5′-ACT AAG CGA GCA CTG CCC AA-3′	5′-GTA AGT CAA GTA GCA TCT GAA GGG TG-3′
Δ40p53	5′-TCC CTG GAT TGG CAG CC-3′	5′-TGG TGG GCC TGC CCT T-3′
Δ133p53	5′-TGA CTT TCA ACT CTG TCT CCT TCC T-3′	5′-GGC CAG ACC ATC GCT ATC TG-3
β-Actin	5′-GTG GGG CGC CCC AGG CAC CA-3′	5′-CTC CTT AAT GTC ACG CAC GAT TTC-3′

### Immunoblotting

Cell line pellets were washed in PBS and then incubated with lysis buffer (1% Triton X-100, 150 mm NaCl, 10 mm Tris (pH 7.4), 1 mm EDTA, 1 mm EGTA (pH 8.0); 0.2 mm Na_3_VO_4_, and 0.2 mm NP-40). 100 μg of protein lysates were resolved using SDS-PAGE electrophoresis and then transferred to nitrocellulose membranes. The anti-p53 antibodies DO-1, DO-7, and 1801 (Santa Cruz Biotechnology, Dallas, TX) were used at 0.8 μg/ml and incubated overnight. Because Δ40p53 lacks the 40 N-terminal amino acids (Fig. 1, *region TAD 1*), it can be distinguished from fl-p53 using a combination of the anti-p53 monoclonal antibodies DO-1 or DO-7 and 1801, which bind to this region. The ECL detection reagent (Amersham Biosciences ECL Prime Western blotting detection reagent, GE Healthcare) was used to detect antigen–antibody complexes. Membranes were imaged using the ChemiDoc MP imaging system (Bio-Rad).

### Immunofluorescence

Cells (5 × 10^5^ per chamber) were seeded into 12-chamber tissue culture slides. The next day, cells were rinsed with ice-cold PBS, fixed with 4% paraformaldehyde, permeabilized with 0.5% Triton X-100, and blocked in 5% BSA. Cells were subjected to immunofluorescence staining with either DO-7 or 1801 anti-p53 antibodies (1:400) and anti-A11 antibody (1:400) and incubated overnight at 4 °C. Cells were then incubated with goat anti-mouse IgG/Alexa 568–labeled anti-rabbit (1:800) or Alexa 488–labeled anti-mouse (1:800) secondary antibodies (Thermo Fisher Scientific) at room temperature for 1 h and finally stained with 4′,6-diamidino-2-phenylindole (DAPI). Cells were then examined using fluorescence confocal microscopy (FV10i-O, Olympus, Tokyo, Japan) and analyzed using FV10-ASW and ISI software.

### Protein expression and purification

The pET15b vectors containing the His-tagged constructs of p53 DBD (Addgene, 24866) and p53 TAD.DBD (Addgene, 24864) were heat shock–transformed into the *Escherichia coli* BL21 (DE3) pLysS strain (Invitrogen, C6060-03), grown at 37 °C in Luria broth to an *A*_600 nm_ between 0.8–1.2, and induced with 1 mm isopropyl 1-thio-β-d-galactopyranoside for 16–20 h at 25 °C. Cell lysis (mass from 2 liters of culture) was performed with 45 ml of lysis buffer composed of 50 mm Tris-Cl (pH 7.4), 150 mm NaCl, 2.5 mm tris(2-carboxyethyl)phosphine (Sigma, 646547) (buffer A) supplemented with 1 mg/ml of lysozyme (Sigma, L6876) and one protease inhibitor mixture tablet (Sigma, S8830). Sonication was performed with cycles of 1 min on and 1 min off for 30–45 min. After centrifugation at 45,000 × *g* for 15 min at 4 °C, soluble fractions were loaded onto a column of nickel-nitrilotriacetic acid–agarose (Qiagen, 30210) coupled to an ÄKTA system. Weakly bound proteins were washed out using buffer A and 25 mm of imidazole, followed by a linear gradient of 150 ml and a target concentration of 500 mm imidazole. Peak fractions were directly loaded onto a Superdex 75 16/600 PrepGrade (GE Healthcare, 28-9893-33) that was previously equilibrated with PBS (137 mm NaCl, 2.7 mm KCl, 10 mm Na_2_HPO_4_, and 1.8 mm KH_2_PO_4_). The concentration of eluted proteins was estimated by absorbance at 280 nm using a molar extinction coefficient of 17,420 m^−1^ cm^−1^ (p53 DBD) and 34,545 m^−1^ cm^−1^ (p53 TAD.DBD). Protein aliquots were stored at −80 °C after addition of 5% glycerol.

### Light scattering and ultracentrifugation

Kinetics monitoring the light scattering of constructs (5 μm) were performed using an ISS-PC1 spectrofluorometer (ISS, Inc.) with excitation and emission at 320 nm at 37 °C and gentle agitation. Ultracentrifugation was performed at 45,000 × *g* for 15 min at 4 °C (Beckman Coulter, Optima TLX) after incubation of 20 μm constructs at 37 °C with 600 rpm agitation (Thermo Scientific, 13687711). Thioflavin T at 25 μm was mixed to assess binding.

### Size-exclusion chromatography

For analytical size-exclusion chromatography, we used a Superdex 200 10/300 column (GE Healthcare, 17-5175-01). Runs were performed in PBS at a flow rate of 0.7 ml/min, and the absorbance was monitored at 280 nm using the ÄKTA Prime System (GE Lifesciences). The column was calibrated previously using thyroglobulin (670 kDa), γ-globulin (158 kDa), ovalbumin (44 kDa), myoglobin (17 kDa), and vitamin B12 (1.35 kDa, Bio-Rad, 151-1901).

### CD

CD was carried out on a Jasco spectropolarimeter (J-1500). Far-UV spectra were recorded from 260 to 200 nm at 25 °C and 75 °C using 0.2-nm steps. Mean residue ellipticity [Θ] in degrees per square centimeter per decimole was calculated using the equation [θ]/*l* × *c* × 10 × *n*, where θ is the measured ellipticity in millidegrees, *n* is the number of peptide bonds in the primary sequence, *l* is the path length in centimeters, and c is the molar concentration. A quartz cell of 2-mm path length was used with protein samples of 10 μm in PBS (140 mm NaCl, 2.7 mm KCl, 10 mm Na_2_HPO_4_, and 1.8 mm KH_2_PO_4_).

### Transmission EM

Images were obtained for TAD.DBD after increasing the temperature from 25 °C to 75 °C at 1 °C/min. Before grid preparation, samples were left for 1 h at room temperature to settle. Samples were applied to previously discharged carbon films on 200-mesh copper grids (EMS, catalog no. CF200-cu) for 1 min, gently dried with filter paper, and stained with 2% uranyl acetate for 5 s. Negatively stained images were acquired on a Philips Tecnai microscope operated at 80 kV at ×46,000 magnification. As DBD samples precipitated heavily, grids were not suitable for imaging.

## Author contributions

N. M. d. S., J. A. M.-D., J. L. S., and E. R. P. G. data curation; N. M. d. S., M. R. R., J. L. S., and E. R. P. G. formal analysis; N. M. d. S., G. A. P. d. O., M. R. R., M. M. P., G. D. d. S. F., L. P. R., J. A. M.-D, J. L. S., and E. R. P. G. investigation; N. M. d. S., M. R. R., M. M. P., G. D. d. S. F., L. P. R., J. L. S., and E. R. P. G. methodology; G. A. P. d. O., L. P. R., J. L. S., and E. R. P. G. validation; G. A. P. d. O., M. R. R., J. L. S., and E. R. P. G. visualization; G. A. P. d. O. and E. R. P. G. writing-original draft; G. A. P. d. O., J. L. S., and E. R. P. G. writing-review and editing; J. A. M.-D, J. L. S., and E. R. P. G. resources; J. A. M.-D, J. L. S., and E. R. P. G. supervision; J. L. S. and E. R. P. G. conceptualization; J. L. S. and E. R. P. G. funding acquisition; J. L. S. and E. R. P. G. project administration.

## Supplementary Material

Supporting Information

## Footnotes

3 The abbreviations used are:

EC: endometrial carcinoma
OD: oligomerization domain
fl: full-length
TAD: transactivation domain
DBD: DNA-binding domain
UC: ultracentrifugation
ThT: thioflavin T
GoF: gain-of-function
DAPI: 4′,6-diamidino-2-phenylindole
aa: amino acids.
