# Supplementary material for: Loss of the p53 transactivation domain results in high amyloid aggregation of the Δ40p53 isoform in endometrial carcinoma cells
Source: J Biol Chem. 2019 Apr 26;294(24):9430–9. doi: 10.1074/jbc.RA119.007566 (PMC6579457; doi:10.1074/jbc.RA119.007566)
Supplement: Supporting Information [file supp_294_24_9430__index.html]

Loss of the p53 transactivation domain results in high amyloid aggregation of the Δ40p53 isoform in endometrial carcinoma cells — Δ40p53 isoform explains p53 aggregation — Loss of the p53 transactivation domain results in high amyloid aggregation of the Δ40p53 isoform in endometrial carcinoma cells — The Δ40p53 isoform explains p53 aggregation — Supporting Information 

# Loss of the p53 transactivation domain results in high amyloid aggregation of the Δ40p53 isoform in endometrial carcinoma cells

## Supporting Information

- Supporting Information (to be published online) - Supporting Figure S1 and Figure S2
