# Supplementary material for: Loss of the p53 transactivation domain results in high amyloid aggregation of the Δ40p53 isoform in endometrial carcinoma cells
Source: J Biol Chem. 2019 Apr 26;294(24):9430–9. doi: 10.1074/jbc.RA119.007566 (PMC6579457; doi:10.1074/jbc.RA119.007566)
Supplement: Supporting Information [file supp_RA119.007566_143147_3_supp_319920_pqwww8.pdf]

Supporting Information

Article Title: The p53 transactivation domain regulates its aggregation and sustain the high Δ40p53 isoform amyloid aggregation in endometrium carcinoma cells

Authors: Nataly Melo dos Santos, Guilherme A. P. de Oliveira, Murilo Ramos Rocha, Murilo M. Pedrote, Giulia Diniz da Silva Ferretti, Luciana Pereira Rangel, José A. Morgado-Diaz, Jerson L. Silva, Etel Rodrigues Pereira Gimba

Figura S1

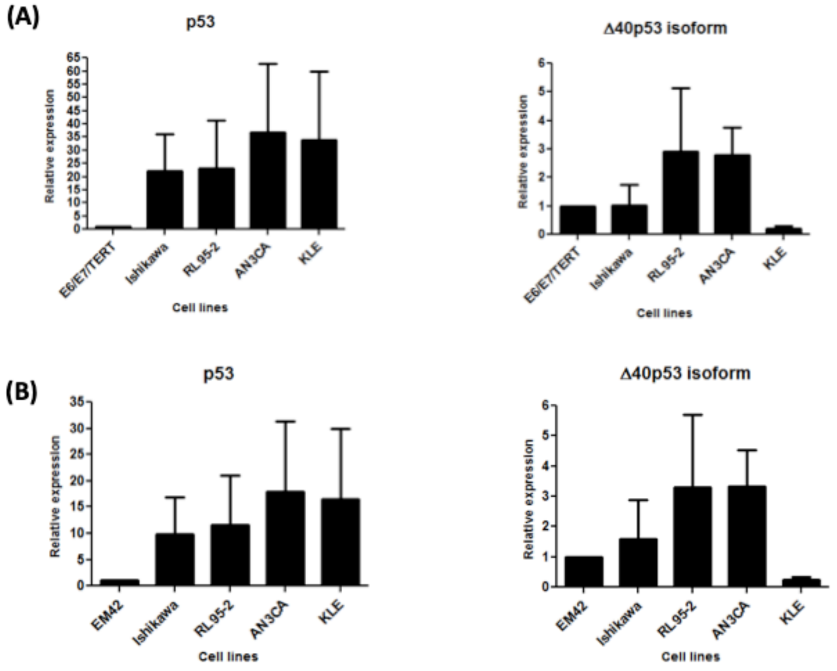

Fig S1: Transcript levels of fl-p53 and Δ40p53 isoforms in endometrial tumor and non-tumor cell lines. Relative transcript levels of fl-p53 (p53) bar graphs on the left) and Δ40p53 (bar graphs on the right) in endometrial tumor cells lines Ishikawa, RL95-2, AN3CA and KLE in relation to E6/E7/TERT (A) or EM42 (B) were determined using quantitative real-time PCR (qRT-PCR) and isoform-specific oligonucleotide pairs (shown on Table 1). β-actin gene was used as a normalization control. Data are representative of three independent experiments using duplicates in each assay. The expression levels of fl-p53 (p53) Δ40p53 in non-tumoral cell lines were used as a reference to calculate the relative expression level of each p53 isoform.

Figure S2

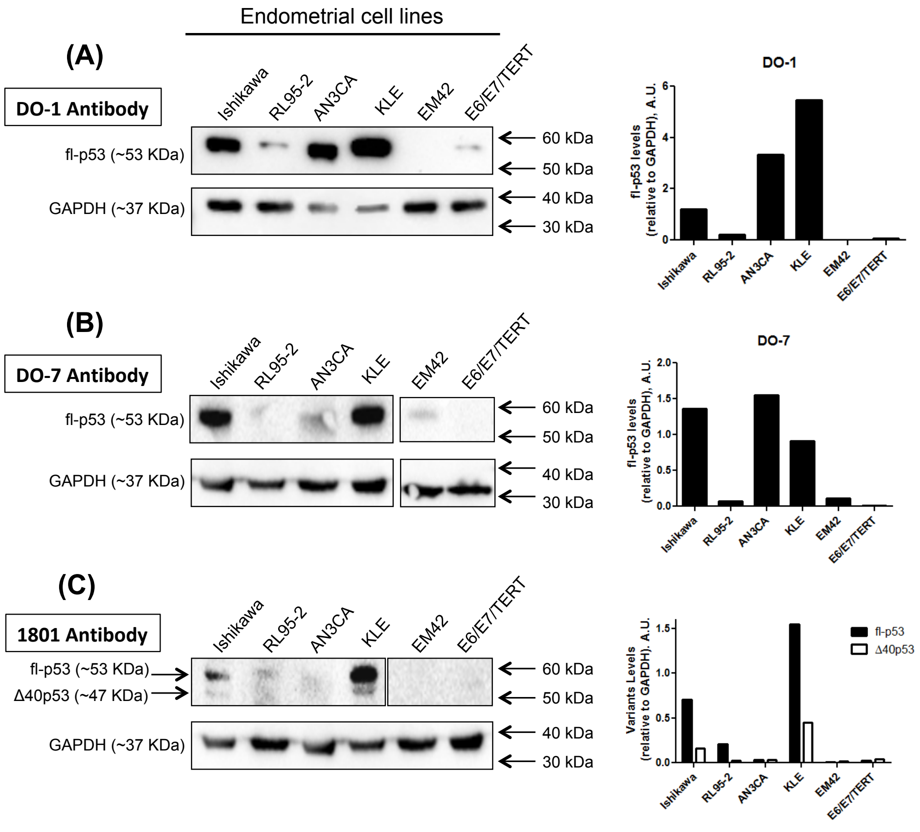

FIGURE S2. Full-length (fl-p53) and Δ40p53 are differentially expressed in endometrial tumor and non-tumor cell lines the major p53 isoforms. Immunoblot analysis of p53 isoforms in endometrial tumor (Ishikawa, RL95-2, AN3CA, and KLE) and non-tumor (E6/E7/TERT and EM42) cell lines using DO-1 (A) ,DO-7 (B) or 1801 (C) anti-p53 antibodies. The fl-p53 isoform (53 KDa) was detected either by DO-1 (A), DO-7 (B), or 1801 (C) anti-p53 antibodies, and a smaller isoform (47 KDa) was only detected by the 1801 anti-p53 antibody. Loading amounts in (A), (B) and (C): 100 µg. The comparison between the variants levels show that the 47 kDa polypeptide is distinct from another detected by DO-1 or DO-7 antibodies. Relative protein levels were quantified by densitometry using arbitrary units (A.U.) and expressed as p53 variant protein levels with GAPDH serving as internal standard and represent data from a single experiment for each blot (n=1) and for this reason statistical analysis has not been performed. Black bars represent fl-p53 (p53) protein levels, while white bars represent Δ40p53 protein levels.
